# Environmentally driven phenotypic convergence and niche conservatism accompany speciation in hoary bats

**DOI:** 10.1038/s41598-022-26453-y

**Published:** 2022-12-19

**Authors:** J. Angel Soto-Centeno, Nancy B. Simmons

**Affiliations:** 1 Department of Earth and Environmental Sciences, Rutgers University, Newark, NJ 70102 USA; 2 Department of Mammalogy, Division of Vertebrate Zoology, American Museum of Natural History, New York, NY 10024 USA

**Keywords:** Phylogenetics, Speciation, Biodiversity, Biogeography, Ecological modelling

## Abstract

Species that are geographically widespread may exist across environmentally heterogeneous landscapes that could influence patterns of occupation and phylogeographic structure. Previous studies have suggested that geographic range size should be positively correlated with niche breadth, allowing widespread species to sustain viable populations over diverse environmental gradients. We examined the congruence of phenotypic and phylogenetic divergence with the environmental factors that help maintain species level diversity in the geographically widespread hoary bats (*Lasiurus cinereus* sensu lato) across their distribution. Genetic sequences were analyzed using multiple phylogenetic and species delimitation methods, and phenotypic data were analyzed using supervised and unsupervised machine learning approaches. Spatial data from environmental, geographic, and topographic features were analyzed in a multiple regression analysis to determine their relative effect on phenotypic diversity. Ecological niches of each hoary bat species were examined in environmental space to quantify niche overlap, equivalency, and the magnitude of niche differentiation. Phylogenetic and species delimitation analyses support existence of three geographically structured species of hoary bat, each of which is phenotypically distinct. However, the Hawaiian hoary bat is morphologically more similar to the South American species than to the North American species despite a closer phylogenetic relationship to the latter. Multiple regression and niche analyses revealed higher environmental similarities between the South American and Hawaiian species. Hoary bats thus exhibit a pattern of phenotypic variation that disagrees with well-supported genetic divergences, instead indicating phenotypic convergence driven by similar environmental features and relatively conserved niches occupied in tropical latitudes.

## Introduction

Understanding the factors that drive population or species distributions and divergence is fundamental in biogeography. Environmental pressures are classically considered to be strong drivers of diversification because they shape patterns of species occurrence and community composition. The successful colonization of a new geographic area with distinct environmental and habitat features may lead to local adaptation, the evolution of specific ecological traits, and reproductive isolation^1–4^. In this mode of ecological speciation, divergence is promoted by selection to cope with different environmental features^5^. Species that colonize an isolated landmass may undergo adaptive radiation, where multiple descendant species exploit niches in dynamic ways and eventually diversify into ecologically distinct forms that differ from their ancestor. This has been shown in famous examples including Caribbean *Anolis* lizards and the Malagasy bird family Vangidae^6,7^. Such examples emphasize selection towards environmental differences and newly available niches in the colonized range leading to rapid evolution^4,8,9^. However, other studies have proposed niche conservatism, or the propensity of species to occupy similar environmental niches over time, as an important driver of speciation^8,10,11^.

Species with high dispersal ability and those that are introduced or invasive can span environmentally heterogeneous landscapes. For example, populations of migratory species may move seasonally across multiple habitats seeking favorable environmental conditions to exploit resources or reproduce. These species have evolved the behavioral, ecological, morphological, and/or physiological traits that allow them to cope with different environments^12,13^. Niche tracking, where a species occupies summer and winter ranges with similar environmental conditions, or niche switching, where a species cycles between different environmental conditions within a range, are two strategies used by migratory birds to overcome the challenges posed by occupying areas with heterogeneous environmental conditions^14^. The bat *Leptonycteris yerbabuenae* also shows niche tracking, and some populations undergo spring migrations that follow the blooming of many dry tropical plants from northwest to southwest Mexico^15,16^. While there is some evidence of niche shifts in invasive plants^17^, studies show that invasive terrestrial plants have a propensity to conserve their ecological niches across geographic space, and species tend to occupy niches within the limits of their native climatic range^11^. For many taxa, climatic niches may be conserved on temporal scales spanning millennia^18,19^. Geographically widespread taxa also confront environmentally heterogeneous landscapes across distinct ecoregions, and may show different patterns of occupation and phylogeographic structure in different regions^20–22^.

Assessments of the ecological factors that help explain variation in mammals are abundant in the literature and many examine genetic and/or morphological variation and the associated environmental features in geographic space^23–27^. Among mammals, bats (order Chiroptera) are an excellent model to study the environmental factors that influence the spatial structure of lineages and speciation^28–30^. Because of their volancy and ability to disperse, many bat species span broad geographic distributions across multiple ecosystems and may even occupy more than one landmass. Some bat families (e.g. Neotropical leaf-nosed bats; Phyllostomidae) are also well known for their high levels of morphological diversity, apparently the result of rapid rates of diversification associated with shifts in ecology and dietary preferences^31–33^. Studies employing barcoding techniques in bats have found high levels of genetic diversity within some genera and species despite little morphological divergence, suggesting possible presence of unrecognized cryptic species^34,35^. However, phenotypic differences have been revealed in many bat genera previously thought to be morphologically homogeneous, highlighting the importance of detailed osteological comparisons^36–39^. Despite the role of environmental and geographic differences in driving genetic and morphological variation, studies documenting diversity in bats, either cryptic or not, often focus on delimiting species or describing variation while providing only a narrow perspective of the factors that aid in species divergence^36,40,41^.

We studied the genetic and morphological variation in hoary bats (*Lasiurus* (*Aeorestes*) *cinereus* sensu lato) and the environmental factors that helped shape this variation across their range. Although some scientists choose to classify hoary bats in a separate genus (*Aeorestes*) from other lasiurines, we follow Simmons and Cirranello^42^ in treating *Aeorestes* as a subgenus of *Lasiurus*. These insectivorous bats have a broad geographic distribution extending from Canada to Argentina and Chile, and they also are the only native mammal in the islands of Hawaii, showing an unparalleled dispersal ability among mammals^43–46^. While classically hoary bats have been thought to represent a single widespread species, recent studies have suggested that *Lasiurus cinereus* sensu lato is actually a complex comprising as many as three species. Hawaiian populations (*Lasiurus semotus*) are distinct from mainland hoary bats, and North and South American hoary bats represent different species (*L. cinereus* and *L. villosissimus*, respectively^47–49^. This three hoary bat species clade is sister to the big red bat, *L. egregius*^47^. Herein, we use phylogenetic and species delimitation approaches to provide a taxonomic backbone against which to test novel hypotheses about the factors that help shape the observed patterns of variation in hoary bats. We first tested the hypothesis that hoary bats constitute three phenotypically distinct groups that are geographically structured and agree with the phylogenetic species limits. Next, we examined our results under the hypothesis that differences found among hoary bat lineages are associated with geographically diverging environmental features using climatic, geographic, and topographic data. We predicted that patterns of genetic and phenotypic differentiation would be congruent showing a strong response to environmental variation that aligns with the occupied environmental niches across each geographic lineage.

## Results

### Phylogenetics and species limits

**Figure 1:**
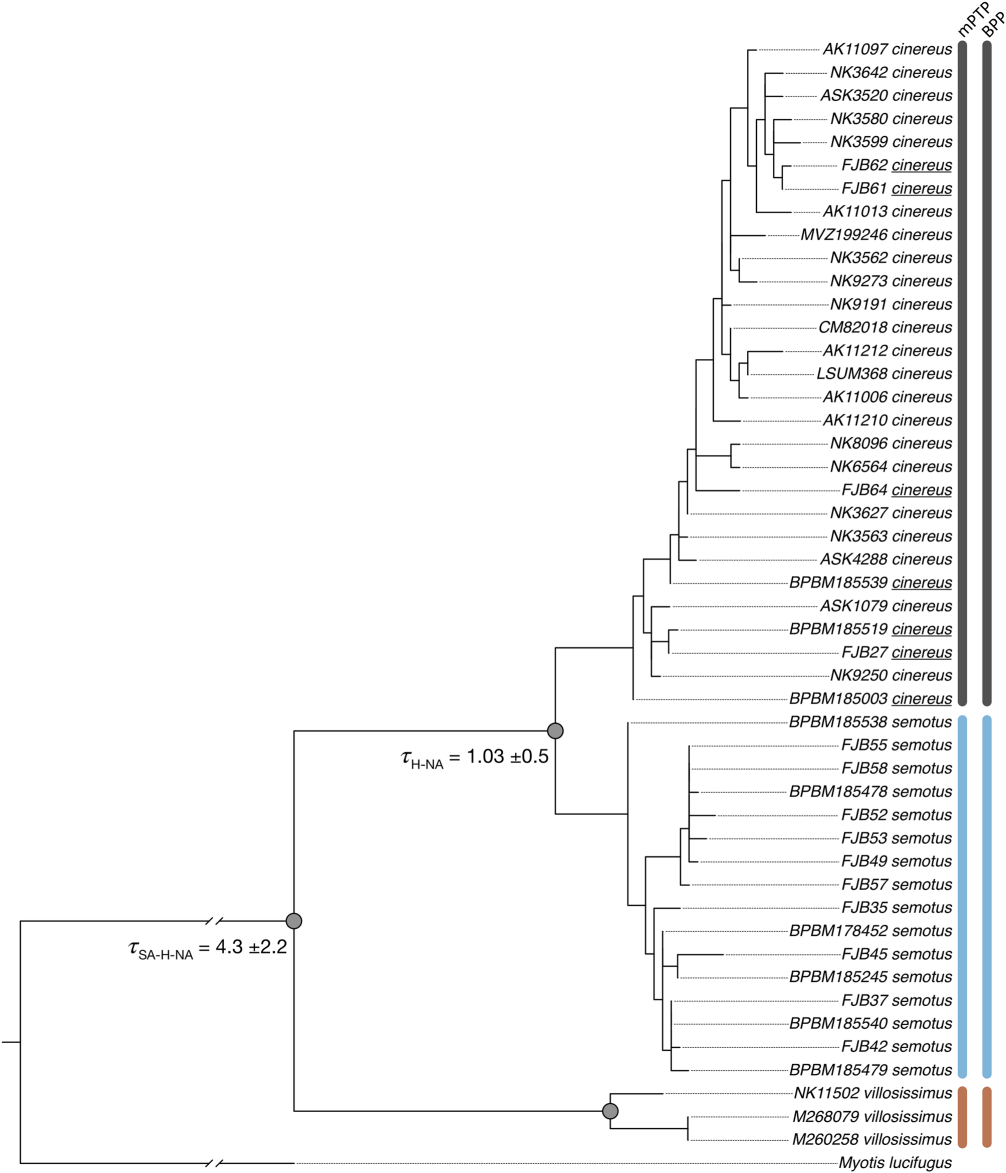
Phylogenetic relationships of Hoary bats (genus *Lasiurus*) based on mitochondrial COI and nuclear RAG2 sequences. Maximum Likelihood (shown) and Bayesian (Fig. S1) analyses recovered identical topologies. Colored circles at nodes represent maximum likelihood Transfer Bootstrap Expectation (TBE) > 70% and Bayesian Posterior Probability (PP) > 0.99 estimated in RaxML-NG and MrBayes, respectively. Divergence times (τ) and 95% HPD were estimated using Bayesian Phylogenetics and Phylogeography (BPP) and shown at species level nodes. Each vertical bar on the right corresponds to summarized results of multi-rate Poisson Tree Process (mPTP) and BPP species delimitation analyses based on the same topology hypothesis and alignment, respectively. Both mPTP and BPP results agree that Hoary bats constitute three species. Terminal taxa represent species names with their respective collection and specimen numbers. Names of specimens of *L. cinereus* resident of Hawaii are underlined. *Myotis lucifugus* (GenBank MG423465.1) was used as outgroup.

Maximum likelihood (ML) and Bayesian phylogenetic analyses confirmed three monophyletic and well-supported clades of hoary bats (i.e. *Lasiurus cinereus*, *L. semotus*, and *L. villosissimus*; Fig. 1 and Fig. S1). The ML and Bayesian topologies resulted in loglikelihoods (− ln) of − 3547.74 and − 3590.37, respectively. Estimation of the divergence parameter (τ) in Bayesian Phylogenetics and Phylogeography (BPP) revealed an early divergence of 4.3 (± 2.2) mya, with *L. villosissimus* as sister to *L. cinereus* and *L. semotus* (Fig. 1) and is congruent with previous findings. The Hawaiian and North American species, *L. cinereus* and *L. semotus*, split about 1 (± 0.5) mya, supporting the hypothesis of Hawaii being colonized from a North American propagule (Fig. 1 in^49^).

The species tree analyses confirmed that hoary bats represent three species (Fig. 1). The three independent methods used as starting delimitation in multi-rate Poisson Tree Process, mPTP (i.e. null model, maximum likelihood, or random) all strongly inferred the three-species tree. BPP analyses rejected the null hypothesis that all hoary bats belong to the same species under all prior setting combinations. The guided (A10) and unguided (A11) runs resulted in the delimitation of three species (Fig. 1). The A11 species tree analysis strongly supported the same topology as ML and Bayesian analyses, with a posterior probability of 0.999 (Table 1).

**Table 1:** Outline of priors and posterior probability results for species delimitation analyses of Hoary bats in BPP. Two analyses were run using a fixed guide tree (A01) and unguided (A11). Two different prior scenarios were examined using five independent runs to ensure parameter convergence based on population size (inverse gamma θ = α, β) and divergence time differences (inverse gamma τ = α, β). For population size, β was estimated from a mutation rate of 2.22 × 10^−9^ substitution/site/year^50^. In the possible alternative topologies presented *C* = *L. cinereus*, *S* = *L. semotus*, and *V* = *L. villosissimus*. In A01 analyses, the initial topology was set to match the relationships obtained from the best supported maximum likelihood tree, indicated by ^a^ (see Fig. 1). P1–3 indicates the posterior probabilities of species delimitation, from one to three species.

Model	Prior (θ)	Prior (τ)	((C, S), V)^a^	((C, V), S)	((S, V), C)	P1	P2	P3
A01	IG(3, 0.008)	IG(3, 0.02)	–	–	–	0	0	1.0
A11	IG(3, 0.008)	IG(3, 0.02)	0.9991	0.0004	0.0004	0	0	1.0^b^

### Phenotypic differentiation

**Figure 2:**
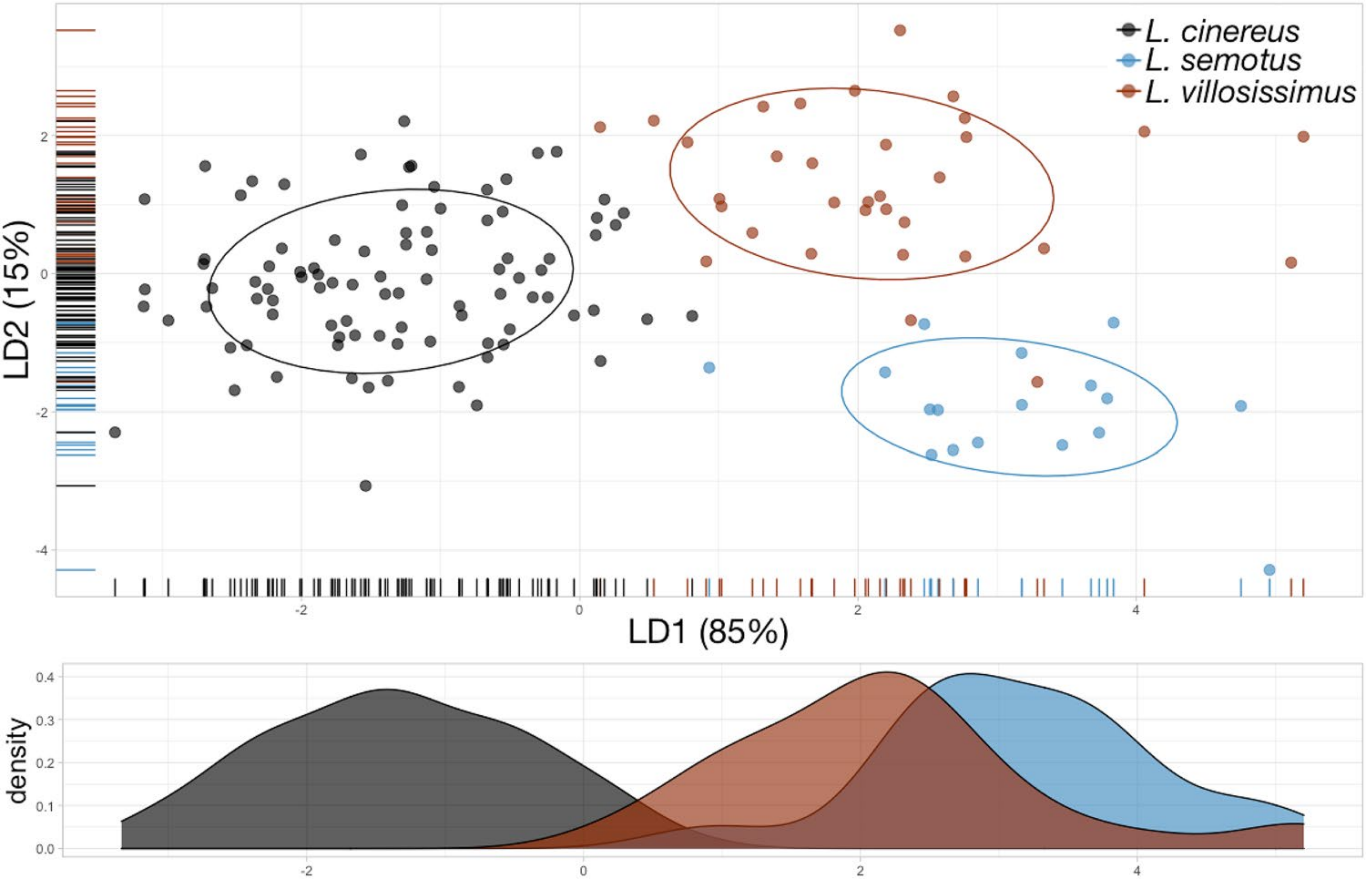
Results from the machine learning Linear Discriminant Analysis (LDA) classifier of phenotypic limits in hoary bats (genus *Lasiurus*). Overall accuracy of the model = 97.2% (95% CI 92.9, 99.2%). Percent group separation described for the first linear discriminant (LD1) was 85 and 15 for the second linear discriminant (LD2). Solid lines represent 68% data ellipses centered at the bivariate mean to visualize phenotypic differences among species. LD1 density values plotted for aid in visualization on the x-axis highlighting closer phenotypic affinities of *L. semotus* and *L. villosissimus*, contrasting with the phylogenetic hypothesis.

Examination of phenotypic variation across geography within *L. cinereus* and at the subspecific level in *L. villosissimus* revealed no intraspecific morphological distinctions (Fig. S3A, S3B). However, despite significant morphological overlap, *L. cinereus* tends to become smaller towards lower latitudes (Fig. S3B). At the species level, analysis of linear discriminants achieved a percent discrimination of 0.85% on LD1 and 0.15% on LD2 (Fig. 2). Our machine learning LDA classifier of phenotypic limits of hoary bats resulted in an accuracy of 97.2% (95% CI 0.929, 0.992), which was significantly better than the no information rate (0.657, *P* < 0.005). The phenotypic classifier could discriminate *L. villosissimus* with 100% certainty. However, some phenotypic overlap was observed in *L. cinereus* with one specimen out of 94 (1% error) incorrectly classified as *L. semotus* (Fig. 2). Phenotypic overlap was even greater among *L. semotus* and *L. villosissimus*, with three specimens out of 17 *L. semotus* (17% error) incorrectly classified as *L. villosissimus* (Fig. 2). All three species of hoary bat were discriminated in the overall morphological space. However, along LD1, *L. cinereus* showed little overlap with the other two species. The craniodental features discriminating the North American species from the rest were associated with the width of the cranium (Table S1; Table S2), specifically the width of the mastoid, the space between the upper canines, and the lacrimal bone width (Table S1; Table S2). Along the same axis, *L. semotus* and *L. villosissimus* are phenotypically similar, contrasting with the proposed phylogenetic relationships. All three species show a greater overlap along LD2, which is characterized primarily by the length of the skull (i.e. the greatest length of skull and length of the lower toothrow).

Results from principal component analysis (PCA) were congruent with those of the LDA classifier (Fig. S2). The proportion of explained variance was 50.4% for PC1 and 7.9% for PC2. While *L. cinereus* was clearly discriminated in morphological space, *L. villosissimus* and *L. semotus* showed considerable phenotypic overlap, contrasting with the proposed phylogenetic relationships. Like LDA, the results from PCA point out to the greatest length of skull, the width between the lachrymal bones, the width of the mastoid, and the length of the rostrum, contributing most to the variability and with *L. cinereus* appearing larger than the other two species.

### Environment–phenotype association

Two variables, precipitation seasonality and precipitation of the driest quarter (bio15 and bio17), had variance inflation factors > 5 (Table 2; Fig. S4). These were removed from multiple regression analyses (Table 2). A marked positive association with phenotypic variation was observed in temperature seasonality (bio4) and latitude (Table 2; Fig. S4). Hoary bat crania tended to be longer and wider at high (i.e. more seasonal and temperate) latitudes than in lower (i.e. more tropical) latitudes (Fig. S4-E, also see Fig. S3B). The phenotypic similarity observed between *L. villosissimus* and *L. semotus* was associated with environmental factors that were more similar between these species than between either and *L. cinereus*. While longitude showed a significant association (*P* = 0.02), this relationship was expected and reflects the isolation of the westernmost hoary bat species in Hawaii.

**Table 2:** Multiple regression model of phenotype–environment associations in hoary bats. The reduced model included six variables (in regular font), of which three showed a significant association with phenotypic variability. Multiple R^2^ = 0.585, adjusted R^2^ = 0.566; F-statistic = 31.2 on 6 and 133 DF; overall model *P* < 2.2^e−16^. Estimated variance inflation factors (VIF) for all variables in the reduced model are below 5, indicating low degree of multicollinearity. Two variables in the full model (bio15 and bio17, shown in italics) exceeded the VIF threshold of 5 and were excluded in the reduced analysis due to collinearity. Regression plots for each variable in the reduced model and variance inflation factors estimates are presented in Supplementary Fig. S4. Significant values are in bold.

Variable	Description	Slope	*t* value	*Pr*(> *|t|*)	VIF
bio4	Temperature seasonality	+	4.035	**9.16**^**e−05**^	3.0
bio5	Max. temperature of warmest month	+	1.109	0.2693	1.1
bio16	Precipitation of wettest quarter	−	1.571	0.118	1.5
bioalt	Elevation	NA	1.908	0.058	1.2
lat	Latitude	+	5.655	**9.13**^**e−08**^	1.8
lon	Longitude	NA	2.216	**0.028**	2.6
*bio15*	*Precipitation seasonality*	NA	*− 0.021*	*0.983*	*6.7*
*bio17*	*Precipitation of driest quarter*	NA	*− 1.723*	*0.087*	*9.1*

**Figure 3:**
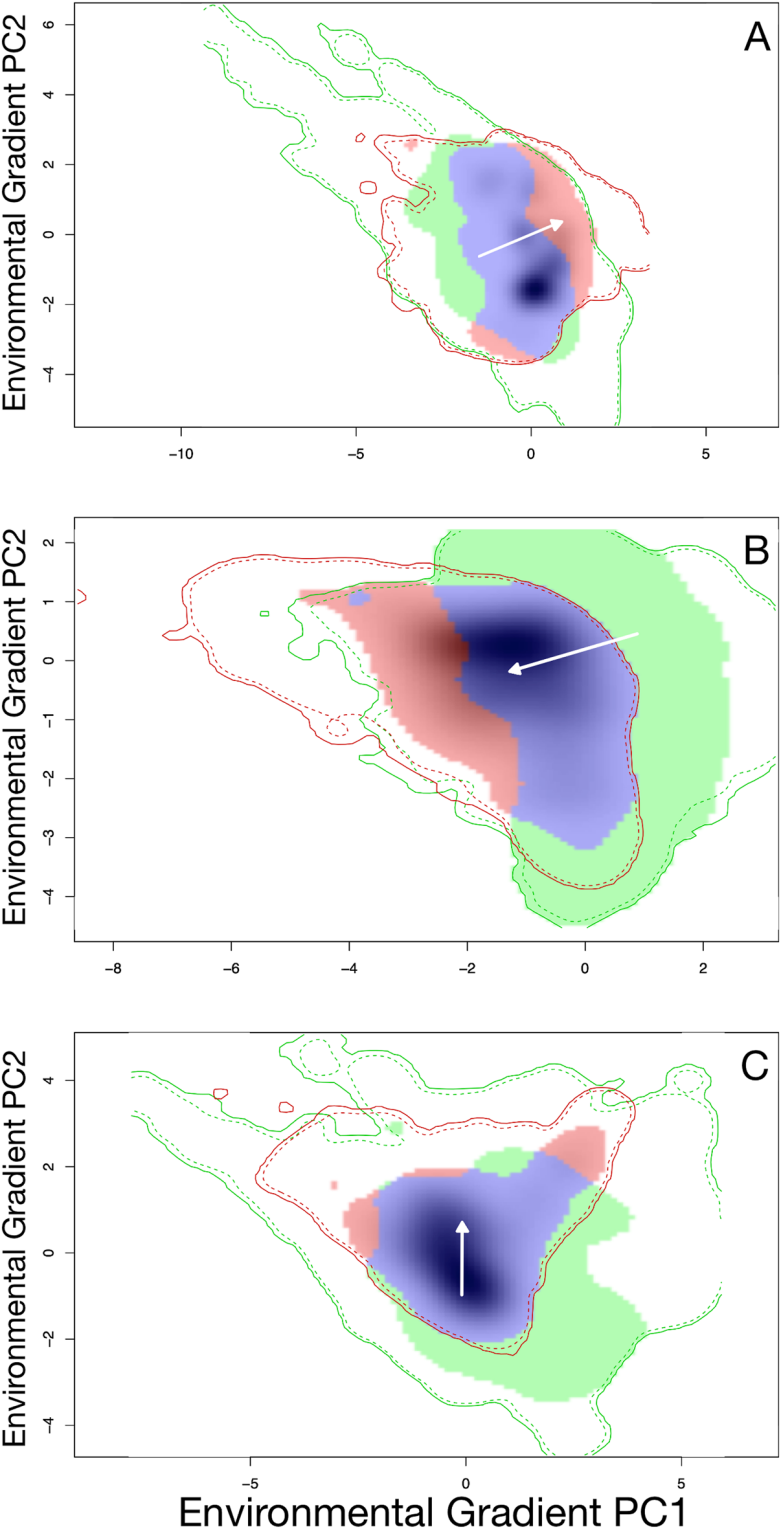
Estimates of niche overlap and shift in hoary bats from their native to their colonized ranges as hypothesized by their phylogenetic divergence. Plots (**A**–**C**) represent pairwise comparisons of niche overlap among species, where (**A**) = *L. villosissimus* vs. *L. cinereus*, (**B**) = *L. cinereus* vs. *L. semotus*, and (**C**) = *L. villosissimus* vs. *L. semotus*. White arrows indicate niche shifts from the centroid of the environmental range of the species set as reference (i.e. in (**A**) = *L. villosissimus*, in (**B**) = *L. cinereus*, and in (**C**) = *L. villosissimus*) to the centroid of the other species range (arrowhead). Blue shading highlights areas of niche overlap between species environmental ranges. Reference species does not include density shading to aid visualization of differences. In all plots, solid contour illustrates the full range (100%) of background environmental space available and dashed contour represents 50% at each spatial scale. Environmental gradients of PC1 and PC2 represent 48% and 19%, 44% and 26%, and 48% and 25% of the variation in plot (**A**), (**B**), and (**C**), respectively.

Evaluation of the environmental niche of each species of hoary bat revealed that a niche shift occurred, as measured by the niche centroid shift, at each point of divergence; that is, between mainland *L. villosissimus* and *L. cinereus,* and between *L. cinereus* to the insular *L. semotus* (Fig. 3A,B). Notably, the magnitude of this shift was smaller and contained within the highest density of overlapping occupied environmental ranges when comparing the niche of *L. villosissimus* to *L. semotus*. This suggests that the occupied niches for these species converged (i.e. are conserved) despite their somewhat more distant phylogenetic relationship (Fig. 3C). Ordination analyses revealed that mainland hoary bats (*L. cinereus* and *L. villosissimus*) have the highest levels of niche overlap in environmental space (Schoener’s D = 0.45; Fig. 3A; Table 3). Low levels of niche overlap were characteristic in pairwise comparisons between mainland vs. island; that is, *L. cinereus* vs. *L. semotus* (Schoener’s D = 0.16; Fig. 3B; Table 3) and *L. villosissimus* vs. *L. semotus* (Schoener’s D = 0.20; Fig. 3C; Table 3). The null hypothesis of niche equivalency was rejected in all pairwise species comparisons, revealing that niches were not identical and supporting our prediction (Table 3). The test of niche similarity was rejected for the bidirectional *L. villosissimus* vs. *L. cinereus* comparisons, suggesting that the measured overlap between species was explained by regional similarities (or differences) in the available suitable habitat between these bats (Table 3). In comparisons of mainland (*L. villosissimus*) with island (*L. semotus*), and island with mainland (*L. cinereus*), niche similarity was not rejected, suggesting that niches were not more similar to each other than expected by chance (Table 3).

**Table 3:** Pairwise niche comparisons among three species of hoary bat. Niche overlap estimates shown for statistical comparisons of niche equivalency and similarity for species combinations *a* and *b*. Niche overlap statistic (Schoener’s *D*) range from 0 (i.e. niches have no overlap) to 1 (i.e. niches are identical). Based on pairwise comparisons of the observed Schoener’s D, *P* values of niche equivalency and niche similarity are presented in their respective columns. Values of niche equivalency and similarity shown in bold denote *P* < 0.05 for niche estimates that are significantly different (i.e. not equivalent) or more similar than expected by chance respectively for each statistic. Niche dynamics show estimated values of expansion (Exp.), stability (Sta.), and unfilling (Unf.) estimated from the data; values shown in bold resulted in *P* < 0.05.

Species				Niche similarity		Niche dynamics		
*a*	*b*	Niche overlap (*D*)	Niche equivalency	*a* to *b*	*b* to *a*	Exp	Sta	Unf
*L. villosissimus*	*L. cinereus*	0.455	**0.001**	**0.007**	**0.046**	0.157	0.842	0.179
	*L. semotus*	0.203	**0.001**	0.079	0.149	0.040	0.959	**0.037**
*L. cinereus*	*L. semotus*	0.168	**0.001**	**0.018**	0.183	0.250	0.749	**0.040**

The niche overlap and similarity results evaluated in light of niche dynamics statistics showed that despite the variability of niche overlap between mainland and island, no significant evidence of niche expansion was observed in all pairwise comparisons (Table 3). This indicates that following colonization into Hawaii, hoary bats occupied climatic conditions close to those observed in their putative ancestral native range (i.e. North America, sensu^43^). Significant niche unfilling was observed between mainland (either *L. cinereus* or *L. villosissimus*) vs. island (i.e. *L. semotus*) lineages, suggesting that not all niches available in the mainland range were occupied relative to the colonized insular range (Fig. 3A–C). Although not significantly different, niche stability between *L. villosissimus* and *L. semotus* was at least 10% higher than in any other pairwise comparison (Table 2).

## Discussion

Species that occupy wide geographic ranges often show variation, either cryptic or not, following heterogeneous environmental gradients^35,36^. The process of speciation itself often requires ecological opportunity and selection towards individual variation adaptive to novel biotic or abiotic environments^51^. Describing and documenting geographic variation, along with the factors that help shape it, is critical to better understand biodiversity. Over the last decade, researchers have emphasized the importance of integrating multiple criteria to provide insight into the process of speciation across space and time, to delimit species, and better understand the units of potential need for conservation^52–55^. Despite these efforts, many studies documenting variation in bats generally exclude assessments of environmental variation and their influence on promoting species limits^56^. In this study, we examined variation in hoary bats, one of the most widely distributed and iconic bats in the New World. We tested the hypothesis that this group is composed of three species and that the observed variation in each is related to heterogeneous environmental conditions associated with geography. Our phylogenetic and species delimitation analyses recovered three well supported lineages with the earliest diverging about 4.3 mya (the South American *Lasiurus villosissimus*, Fig. 1) and sister to the North American *L. cinereus* plus Hawaiian *L. semotus* (ca. 1 mya; Fig. 1). These results supported hypotheses based on previous studies^47,49^, although those studies did not explore phenotypic variation among groups nor provide context to explain what environmental factors could have promoted this divergence.

We observed congruency in two (LDA and PCA) machine learning approaches examining the phenotypic features of the three hoary bat species (Fig. 2 and Fig. S2). While the three-species hypothesis is supported based on phenotypic information, two patterns were evident. First and overall, *L. semotus* is phenotypically more similar to *L. villosissimus* than to *L. cinereus*, a pattern that contrasted with the phylogenetic relationships (Fig. 1). Second, careful examination of the confusion matrix from the LDA showed some misclassification of individuals among species based on phenotypes alone. We emphasize, however, that this was unlikely due to a problem with the classification models, but rather that it reflects testable patterns of morphological similarity. Specifically, *L. cinereus* shows a gradient of sizes across North America, with smaller individuals found in Mexico (Fig. S3B); these smaller individuals appear phenotypically similar to *L. semotus* (Fig. 2). Hoary bats are thought to have colonized Hawaii twice from mainland North America, and insular *L. cinereus* and *L. semotus* exist sympatrically (Fig. 1, and^43,45^). Thus, phenotypic similarity between these two species could reflect a signal of the geographic source of this colonization. Further work to examine the colonization of Hawaii by hoary bats should expand genetic sampling in Mexico to determine the potential of this region as source, given the observed patterns of phenotypic similarity. Finally, we interpret the 17% error rate of *L. semotus* classified as *L. villosissimus* to directly represent the similarity between these two species showing what constitutes a tropical phenotype for this clade (Fig. 2).

Hoary bat groups examined via multiple linear regressions indicated an association between phenotypic and some environmental variables (Table 2; Fig. S4). The overall strongest associations were observed between bat phenotypes and temperature seasonality (bio4) and latitude (Fig. S4-A,E). These two features suggest a pattern of environment-driven phenotypic similarity between *L. semotus* and *L. villosissimus*. Both species, in contrast to the larger *L. cinereus*, tended to be smaller in zones where the temperature seasonality is lower (i.e. less seasonal) and at lower latitudes, in other words, in areas with more tropical conditions. The convergence in specific ecological niche occupancy (see below) and phenotypic variation observed within and among groups could potentially be drivers of size differences that reflect a pattern that adheres to Bergmann’s rule^57^.

Our niche analysis approach showed moderate to low overlap in occupied niches and indicated that niches among species are not interchangeable (i.e. we rejected niche equivalency; Table 3). This was perhaps not surprising given the highly heterogeneous environmental conditions across the Americas and wide distribution of hoary bats. Overall, we observed that each species of hoary bat occupies the available niche space in different ways as exemplified by the magnitude of shifts and the proportion of occupied niches. While there was a slightly higher level of niche overlap between the Hawaiian *Lasiurus semotus* and South American *L. villosissimus*, overlap values were similarly low in mainland (i.e. *L. cinereus* or *L. villosissimus*) vs. island (i.e. *L. semotus*) comparisons (Table 3). Niche similarity statistics indicated that the overlap between species cannot be explained by the similarities (or differences) in the available habitat, except for comparisons involving *L. cinereus*. However, the niche expansion statistic, although not significant (*P* = 0.10), indicated that a smaller proportion of the niche of the *L. villosissimus* niche did not overlap with that of *L. semotus* (0.04, Table 2), compared to the proportion shared between the more closely related *L. cinereus* vs. *L. semotus* (0.25; Table 2). Furthermore, the significant niche unfilling statistic between both mainland species vs. *L. semotus* suggested that nearly equal proportions of the mainland niche did not overlap with the insular niche (0.03 vs. 0.04 in *L. villosissimus* and *L. cinereus*, respectively; Table 2). When examined in terms of the magnitude of niche shifts, as evidenced by the vector quantifying the change between the centroid of the occupied niches of each species, we observed a smaller niche shift between *L. villosissimus* and *L. semotus* compared to that of *L. cinereus* and *L. semotus* (Fig. 3B,C). Furthermore, this vector was completely contained within the area of overlap, emphasizing niche similarities between these species. Taken together, these results suggest that despite the overall differences in which each species use the available niche space, the magnitude of niche similarity between *L. semotus* and *L. villosissimus*, two phylogenetically distant species, is higher than between *L. semotus* and *L. cinereus*. We suspect that phenotypic similarities between *L. semotus* and *L. villosissimus* could likely result from convergence in similar environmental conditions. This is plausible given the South American origin of *L. egregius*, the sister taxon to hoary bats^47^, and the closer relationship of *L. cinereus* and *L. semotus* (this paper, and^43,47^). However, more data is needed to fully disentangle the possibility that *L. cinereus* is phenotypically divergent from *L. semotus* and *L. villosissimus*.

Wiens^8^ proposed that closely related species should show similarities in ecological niches over evolutionary timescales, and that failure to adapt to novel environmental conditions in a newly colonized place is a key factor in initially isolating populations and creating new lineages. For hoary bats, we observed that even when available (i.e. background) environmental conditions are congruent, occupied ecological niches show a certain degree of divergence (Fig. 3; Table 2). Assessment of niches in environmental space showed that a high proportion of niche areas occupied in North America (*L. cinereus*) represent conditions also available but unoccupied in South America by *L. villosissimus* (i.e. the niche represented by the red shades within the solid and dotted green lines in Fig. 3A). Similarly, a high proportion of niche areas occupied by hoary bats in Hawaii (*L. semotus*) represent conditions also available but unoccupied in North America by *L. cinereus* (i.e. the niche represented by the red shade within the solid and dotted green lines in Fig. 3B). This suggests that other factors may be contributing to the dynamic way that niches are occupied. Notably, in modern times, hoary bats are the only terrestrial mammal native to Hawaii^45^. This could have promoted speciation if colonizing lineages occupied a novel portion of niche space in the colonized insular range. We hypothesize that successful colonization of Hawaii by hoary bats could have been facilitated by lack of competition followed speciation due to lack of geneflow. This could have led to ecological release in an area that lacked the biotic factors present in the native range despite having high abiotic niche similarity.

Recent studies examining the spatial association of morphological variation in bats have suggested that differences in environmental conditions may be drivers of phenotypic divergence. Morales et al.^58^ investigated genetic and morphological variation in relation to geographic and environmental factors in populations of Brazilian free-tailed bats (*Tadarida brasiliensis*). While morphology in Brazilian free-tailed bats seems to be primarily driven by geography and less strongly by environmental factors, the observed genetic variation was attributed to genetic drift. In the Pallas’ long-tongued bat (*Glossophaga soricina*), Cahahorra-Oliart et al.^36^ documented four cryptic species with morphological features of the skull that apparently converge among island vs. mainland groups, which they attributed to the similarities in environmental features. Many other studies documenting cryptic speciation in bats typically rely only on genetic groupings from barcode genetic data or present complete congruence of genetic and morphological data (e.g.^34,35,59^). Our hoary bat study revealed phenotypic groupings that disagree with strongly supported phylogenetic relationships (Figs. 1 and 2). Superficial examination of these data suggests ecological speciation associated with the mainland vs. insular landscapes. Nonetheless, this hypothesis could only be examined by testing for niche differences in environmental space as the tiebreaker between the contrasting genetic and phenotypic results.

Does the Hawaiian hoary bat deserve taxonomic recognition at the species level? Ziegler et al.^45^ described a now extinct second bat species from Hawaii (*Synemporion keana*: Vespertilionidae) and comparative radiocarbon (^14^C) dates between remains of this bat and of *L. semotus* (listed as *L. cinereus semotus*, op cit.) suggested that the two species were likely sympatric over the last 10 kya. In their description, the authors recognized the unique morphology of *L. semotus*^48^, but pointed out the low mitochondrial sequence divergence (ca. 2% in cytochrome *b*) and highlighted that the Hawaiian *Lasiurus* populations resulted from two recent dispersal events, with a first colonization ca. 10 ky and a second one only ca. 800 y^43^. These observations led them to retain the Hawaiian hoary bat as a subspecies, rather than recognizing it as a distinct species, due to a lack of evidence^45^. However, this omitted consideration of the deeper ca. 1 my phylogenetic divergence observed between *L. cinereus* and *L. semotus*^47^ (and this paper). Our analyses integrating coalescent phylogenetic and machine learning phenotypic species limits with ecological niche dynamics, shed light into how these lineages are maintained, and provided further evidence for the recognition of *L. semotus* as a unique species endemic to the Hawaiian Islands.

## Materials and methods

### Gene sequences

Available hoary bat sequences were obtained for the mitochondrial cytochrome oxidase I (COI; 657 bp) and the nuclear Recombination-activating gene 2 (RAG2; 706 bp) from previously published records of hoary bats (*Lasiurus cinereus*, *L. semotus*, and *L. villosissimus*) in^43,47,49^. We reduced bias in the phylogenetic analysis by creating a dataset with minimal sequence gaps, which resulted in 56 individuals including COI and RAG2 genetic markers. Identical sequences (N = 7) were removed before analysis. The little brown bat (*Myotis lucifugus*) was used as outgroup in phylogenetic analyses. Sequences were aligned using the Muscle algorithm in Geneious Prime v2020.1.2 (Biomatters Ltd.). The final alignment file is available in fasta format and archived in 10.5281/zenodo.6946172. Genbank accession numbers of gene sequences used are listed in Table S3.

### Phylogenetic inference and species delimitation

Phylogenetic relationships among putative *L. cinereus*, *L. semotus*, and *L. villosissimus* lineages were estimated following a maximum likelihood approach implemented in RAxML-NG^60^. This analysis consisted of a GTR + FO + G4m model of substitution and run over 50 independent tree searches using 25 random and 25 parsimony-based starting trees to produce the best scoring topology. We replicated this analysis in three independent inference runs. To ensure that the likelihood surface of tree space was thoroughly explored, we compared loglikelihoods by computing the topological Robinson-Foulds (RF) distances^61^. Branch support was evaluated using 2000 MRE-based bootstrap replicates^62^, where an auto MRE cutoff of 0.03 was used to automatically assess if sufficient replicates were performed. Transfer bootstrap expectation (TBE^63^) metrics from all bootstrap replicates were used to summarize branch support and mapped onto the best topology. TBE offers a good reliability to recover branch support because it is based on the minimum transfer distance between a given branch and any other branch in the bootstrap replicate tree^63^. We also estimated phylogenetic relationships using Bayesian inference in MrBayes 3.2^64^. For this approach, we used a GTR + I + G model of substitution in two 1 × 10^6^ generation runs with 4 Markov chains, sampled every 2 million generations. Convergence was evaluated by the average standard deviation of split frequencies (< 0.01) and by examining log-likelihood scores in Tracer v1.7^65^ to assess stationarity after the first 10% of trees were discarded as burn-in. A 50% majority rule consensus tree was generated to calculate posterior probabilities (PP). TBE and PP were mapped onto the maximum likelihood phylogeny to assess overall congruence of support.

To test the genetic hypothesis that hoary bats are three species, we evaluated species limits using two species delimitation methods. First, we used the single locus maximum likelihood coalescent approach multi-rate Poisson Tree Process, mPTP^66^. The mPTP analysis was run in the server https://mcmc-mptp.h-its.org/mcmc and used the non-ultrametric tree for the focal taxa produced in RAxML-NG. The MCMC runs included 10 × 10^6^ generations that were sampled every 10 thousand after a 10% burn-in. Three separate analyses were set with different starting delimitation models: null model (i.e. all lineages considered as constituting one species), maximum likelihood model (i.e. MLE based delimitation), and random model (i.e. random delimitation). In each analysis, the option *--multi* was used to include the intra-specific differences among rates of coalescence with a minimum branch length of 0.001. Second, we used the multi-locus coalescent-based approach Bayesian Phylogenetics and Phylogeography, BPP v4.2.9^67^ following the guidelines of Flouri et al.^68^. An initial species tree estimation (A01 analysis) was run using the fixed maximum likelihood topology as a guide, a diffuse prior for θ in which ⍺ = 3 and β was estimated from the standard mutation rate for mammals of 2.22 × 10^−9^ substitution/site/year^50^. The approach of using a diffuse prior of τ included ⍺ = 3 and adjusting β ranging from 0.02 to 0.002 on multiple test runs to ensure convergence of the mean onto previously published divergence estimates of hoary bats by Baird et al.^47^. To compare the consistency of the A01 results, we performed an unguided delimitation analysis (A11 analysis) and examined if the same species limits were recovered. Each run consisted of an MCMC chain of 2 × 10^6^ generations, sampling every second generation with a 10% burn-in. All species delimitation analyses were tested in five independent runs to ensure the reliability of results. Convergence was determined by examining the loglikelihood values of each run using Tracer v1.7^65^. Finally, to estimate the divergence parameter (τ) under the multiple species coalescent, we fixed the species tree and ran an independent A00 analysis.

### Morphological data and analyses

To evaluate phenotypic variability among hoary bat species, we examined 140 vouchered museum specimens from localities independent from those in the genetic dataset but encompassing the same geographic regions and with an improved sampling and coverage. Specimens from putative *Lasiurus cinereus* sensu stricto included material from Canada, Mexico, and United States (N = 90), *L. semotus* from Hawaii (N = 17), and *L. villosissimus* included specimens from Argentina, Chile, Colombia, Peru, Uruguay, Venezuela, and Galápagos Islands (N = 33). We measured 16 craniodental characters (Table S1) to the nearest 0.01 mm from each specimen using digital calipers (Mitutoyo, Japan). Some measurements could not be taken from all specimens due to damage and fragmentation of specimens. To overcome the challenge of missing data and maximize the information content of our phenotypic data, we partitioned the data per species and geographic locality and then imputed all missing data using multivariate imputation by chained equations approach in the R package ‘*mice*’^69^*,* ensuring that missing data per partition did not exceed 40% of each character before imputation^70^. To avoid introducing potential biases of rendering traits non-independent or reducing differences between populations^71^, a size correction to morphological measurements among populations was not applied. Furthermore, to rule out the influence of intraspecific size variation, we examined three of the four putative subspecies of *L. villosissimus* (see^72,73^). Because no known subspecific designations are recognized in *L. cinereus*^72,73^, geography was used as a proxy for examining variation. All data wrangling and analyses were done in the R statistical environment^74^ and the final dataset used in this study is available as a text file and archived in 10.5281/zenodo.6946172; a full list of specimens examined, catalog numbers, and institutions is available as supplementary material (Table S4). All morphological data were log-transformed, scaled, and centered before ordination analyses.

We used geography and previous genetic information to classify hoary bat specimens into the phylogenetic grouping of Baird et al.^47^ and tested the hypothesis that each forms a distinct lineage based on morphology. We explored the degree of phenotypic divergence among the three species of hoary bat using linear discriminant analysis (LDA), a supervised machine learning classification algorithm, in the R packages ‘*caret*’ v.6^75^ and ‘*MASS*’ v.7.3^76^. The classification models were trained using a fixed 75% random data partition and then tested using the remaining 25% of the data implementing a k-fold cross validation approach of five replicates. The LDA results are interpretated and visualized similarly to principal components analysis (PCA). A confusion matrix was computed to confirm the accuracy of the LDA model (i.e. how well the classifier assigned each species to the correct group), and then we evaluated whether the overall accuracy rate was statistically greater than the no-information rate^77^. Phenotypic species limits were then examined in a two-dimensional plot of the first two linear discriminants and 68% data ellipses centered at the bivariate mean were used to visualize phenotypic differences. Finally, we also examined the morphological data at the species level using principal component analysis (PCA; *prcomp* function in R) to confirm phenotypic groups. Plots of the first two components (i.e. PC1 and PC2) were used to evaluate the phenotypic variability among the three species of hoary bat.

### Spatial and environmental data

Location data for all 140 vouchered specimens used in the phenotypic analyses were georeferenced to a resolution of 0.01° (1.11 km). This provided a direct spatial dataset linked to the phenotypic description of each individual bat. To supplement this dataset and compute niche dynamics of each species of hoary bat, we used all available georeferenced specimen records (N = 1033 *L. cinereus*, N = 90 *L. semotus*, and N = 51 *L. villosissimus*) from the Global Biodiversity Information Facility (GBIF; http://www.gbif.org). Niche estimation is dependent on the choice of extent of the study background areas^9^. Given the high dispersal potential of hoary bats, we selected background areas by creating a minimum convex polygon around all known presence localities for each species, added a buffer of 10° (ca. 690 km) around each, generated evenly spaced points with a 5 km distance, and extracted climate information for all points. All spatial data was assessed for accuracy and quality before analyses, and all dubious records obtained from the GBIF data (N = 237) were discarded.

Environmental and elevation data were obtained from WorldClim (http://www.worldclim.org) at a spatial resolution of about 5 km (2.5 arc min^78^). All variables were cropped to the regional extent occupied by each hoary bat species using the ‘*raster*’ package in R^79^. To correct for multicollinearity among the 19 WorldClim variables and elevation, we performed a Pearson Correlation in R (*cor* function) and excluded variables with a correlation coefficient > 0.75^19,80,81^. The climate variables retained included Temperature Seasonality (bio4), Max Temperature of Warmest Month (bio5), Precipitation Seasonality (bio15), Precipitation of Wettest Quarter (bio16), Precipitation of Driest Quarter (bio17), in addition to elevation (bioalt; Supplementary Fig. S5).

### Relationship between morphology and environment

We performed a multiple linear regression to assess the factors that influence the maintenance of phenotypic variation in hoary bats. This approach was aimed at examining the relative effects of environmental, topographic, and geographic variables on phenotypic variation. Environmental variables, such as those used here, often show high correlation^19,81^. To further reduce colinearity, we used variance inflation factors (VIF) to identify correlated variables from a full multiple linear regression model. Variance inflation factors estimate how the variance of the of the computed regression coefficient increases due to variable colinearity^82^. Generally, a VIF value ≤ 5 indicates low levels of correlation among variables^82^. A reduced multiple linear regression was run after excluding variables with VIF > 5.

Assessment of phenotypic variation using LDA, a supervised machine learning technique, maximizes the separation between known groups established a priori to provide individual classification^83^. Thus, LDA would be a biased measure of phenotypic differentiation in a regression analysis. Instead, we used the results of the first component from PCA to provide a measure of phenotypic variability. PCA is an unsupervised method designed to find patterns in the data without reference^84^; thus, removing the a priori group identity bias. To directly link phenotypic and environmental data, in this analysis we used the 140 vouchered hoary bat specimens with morphological data and extracted environmental data for each. We first ran a full multiple linear regression model using the *lm* function in R and including all variables (i.e. altitudinal: elevation in meters; environmental: bio4, bio5, bio15, bio16, bio17; geographic: latitude and longitude) compared to phenotypic variability (i.e. PC1). Then, used VIF to remove correlated variables (i.e. bio15 and bio17). Finally, an improved multiple linear regression model was calibrated using the subsampled variables to examine the relative effects of environmental and geographic features on phenotypic variation.

To decompose and quantify the niche of each species of hoary bat, we used the Centroid shift, Overlap, Unfilling, and Expansion (COUE) scheme^85^. This framework, implemented the R package ‘*ecospat*’ v.3.2^86^, uses an ordination based approach that can overcome biases associated with quantifying niche dynamics in geographic space, with differential sampling efforts and/or spatial resolution^87,88^. The analysis was performed using the entire spatial dataset (N = 1174 observations and ca. 119,000 background localities), and five uncorrelated environmental variables (i.e. bio4, bio5, bio15, bio16, bio17) and elevation. Phylogenetic information from the species delimitation analysis was used to do three pairwise comparisons (South America vs North America, South America vs Hawaii, and North America vs Hawaii), and computed the degree of niche overlap (i.e. Shoener’s D^89,90^), and niche dynamics statistics^86,87^. Niche equivalency tests were performed to assess whether the ecological niches of hoary bats are significantly different or interchangeable from each other^89^. This analysis consisted of pairwise comparisons of Shoener’s D overlap values to a null distribution based on 1000 random replicates^86^. Equivalency was determined if the observed Shoener’s D overlap value was significantly lower than overlap values in the null distribution. The niche equivalency tests only assessed whether each pair of species were identical in environmental niche space that exists in the exact localities without considering the surrounding space. Thus, we performed 1000 replicates of niche similarity tests to assess if the environmental niche space for each pair of species is more similar than expected by chance^89^. Finally, niche statistics of centroid shift, overlap, unfilling, and expansion were calculated to disentangle the dynamic way in which niches are occupied among the three species of hoary bat.

## Supplementary Information

Supplementary Information.

## Data Availability

Gene sequences for loci used in phylogenetic reconstruction and species delimitation are available in the NCBI GenBank. A complete list of relevant NCBI accession numbers for each individual is available in the Supplementary Table S3. Sequence alignment, tree files, and a text file including all specimen information (i.e. museum collection numbers, georeferenced localities, all morphological measurements, and environmental data to use in phenotypic and environmental analyses) are deposited in 10.5281/zenodo.6946172.
