# Supplementary material for: Environmentally driven phenotypic convergence and niche conservatism accompany speciation in hoary bats
Source: Sci Rep. 2022 Dec 19;12:21877. doi: 10.1038/s41598-022-26453-y (PMC9763480; doi:10.1038/s41598-022-26453-y)
Supplement: Supplementary file 1 — Supplementary Information. [file 41598_2022_26453_MOESM1_ESM.docx]

Environmentally driven phenotypic convergence and niche conservatism accompany speciation in hoary bats

J. Angel Soto-Centeno^1,2^*, and Nancy B. Simmons^2^

^1^ Department of Earth and Environmental Sciences, Rutgers University, Newark, NJ 70102

^2^ Department of Mammalogy, Division of Vertebrate Zoology, American Museum of Natural History, New York, NY 10024, USA

*Corresponding author: angelo.soto@rutgers.edu

Supplementary Figure S1: Bayesian phylogenetic relationships of Hoary bats (*Lasiurus cinereus* sensu lato) based on mitochondrial COI and nuclear RAG2 sequences and produced in MrBayes 3.2. Topology is identical to that one recovered using maximum likelihood (ML, see Figure 1 in article). Colored circles at nodes represent Bayesian Posterior Probability (PP) > 0.99 estimated in MrBayes. Note: Individuals labeled with asterisks, FJB60 and FJB63, are absent from ML analysis because these have identical sequences.


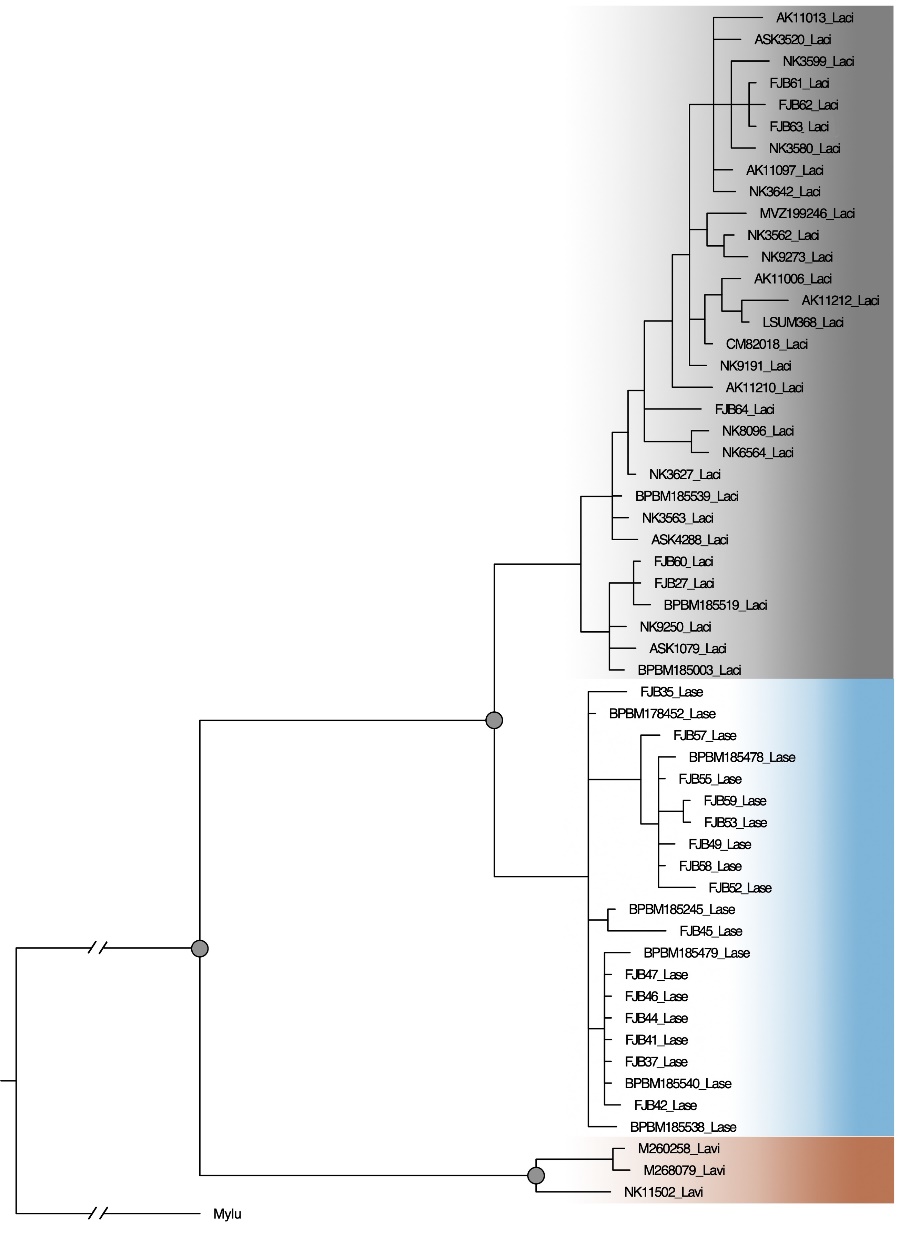


*

*

*L. villosissimus*

*L. cinereus*

*L. semotus*

Supplementary Figure S2: Results from principal component analysis on the phenotypic variability of hoary bats. The explained variance of PC1 = 50.4% and PC2 = 7.9%. Solid lines represent 68% data ellipses to help visualize the phenotypic overlap among species. Notably, *Lasiurus cinereus* can be discriminated from *L. villosissimus* and *L. semotus* along PC1. Contrary to the known phylogenetic hypothesis (see Fig. 1), *L. villosissimus* and *L. semotus* show high overlap in morphological space, indicating that these species are phenotypically more similar to each other.


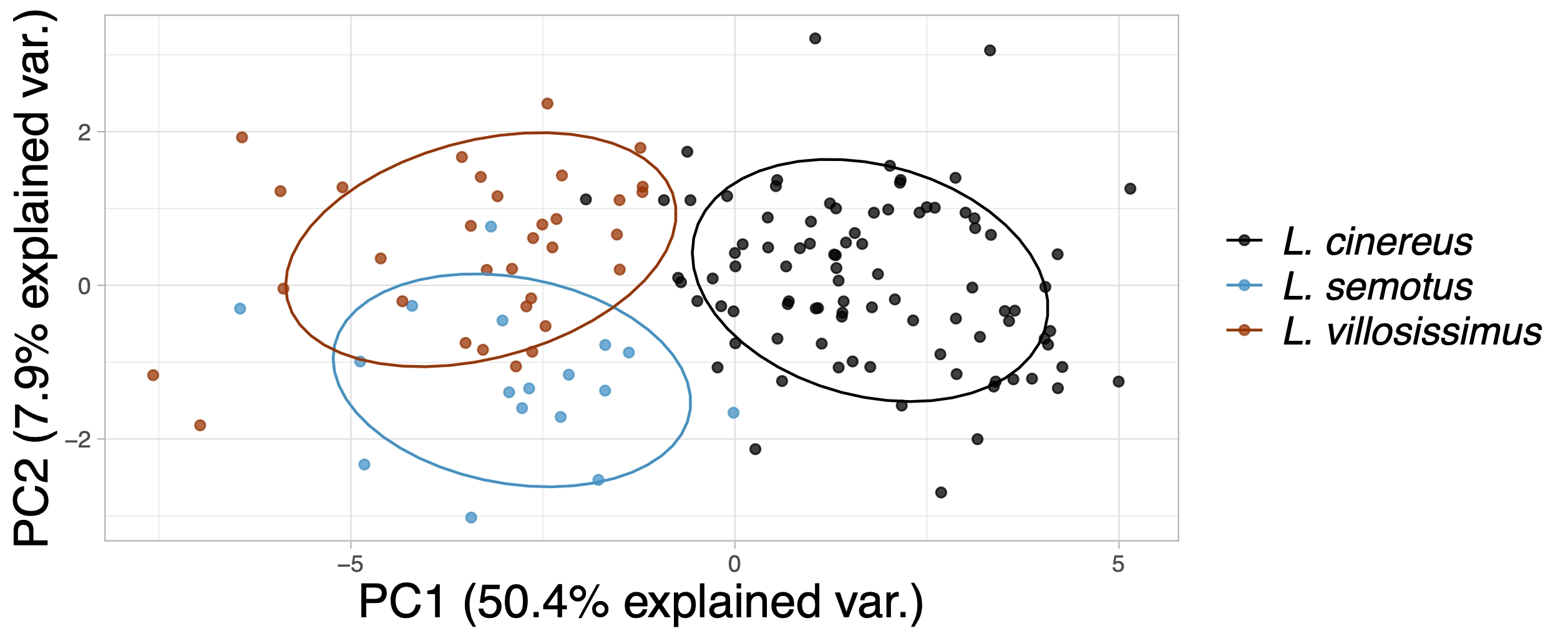


Supplementary Figure S3A. Pairwise comparison results from principal component analysis on the intraspecific phenotypic variability of *Lasiurus villosissimus* from South America. *L. v. grayi* Tomes, 1857 = Chile; *L. v. pallescens* Peters, 1870 = Colombia and Venezuela; *L. v. villosissimus* Geoffroy Saint-Hilaire 1806 = Peru to Bolivia, Paraguay, south Brazil, Uruguay, and Argentina. The subspecies *L. v. brasiliensis* Pira, 1904 was not examined. Individuals from Galápagos do not have subspecific designation but were separated to better understand their respective variability against other geographic groups. Cumulative proportion of variance of PC1 to PC5 is 73%. Solid lines represent 68% data ellipses.


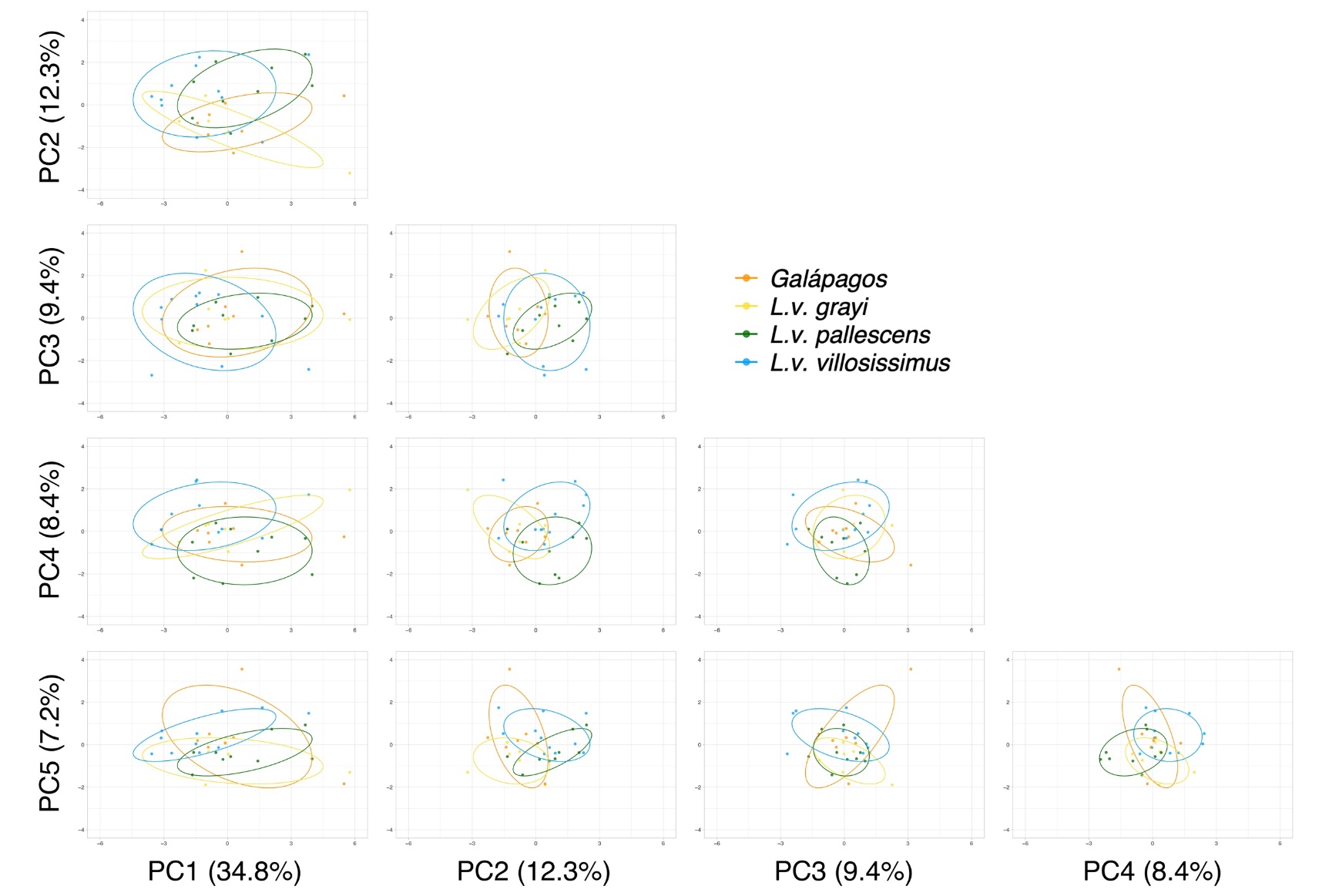


Supplementary Figure S3B. Intraspecific phenotypic analysis of *Lasiurus cinereus*. Because this taxon does not have recognized subspecies, variation was examined using geography (i.e. Canada, Mexico, and United States) as a proxy. A = Linear discriminant analysis showing high phenotypic overlap across geography. Solid lines represent 68% data ellipses. B = Linear regression showing a significant association of phenotypic variation and latitude (R^2^ = 0.482, F-statistic = 81.95 on 1 and 88 DF, P = 3.196^e-14^). C = Boxplots of character measurements representing the top four coefficients of linear discriminants from A. Despite significant overlap, there is a tendency for *L. cinereus* to becoming smaller towards lower latitudes.


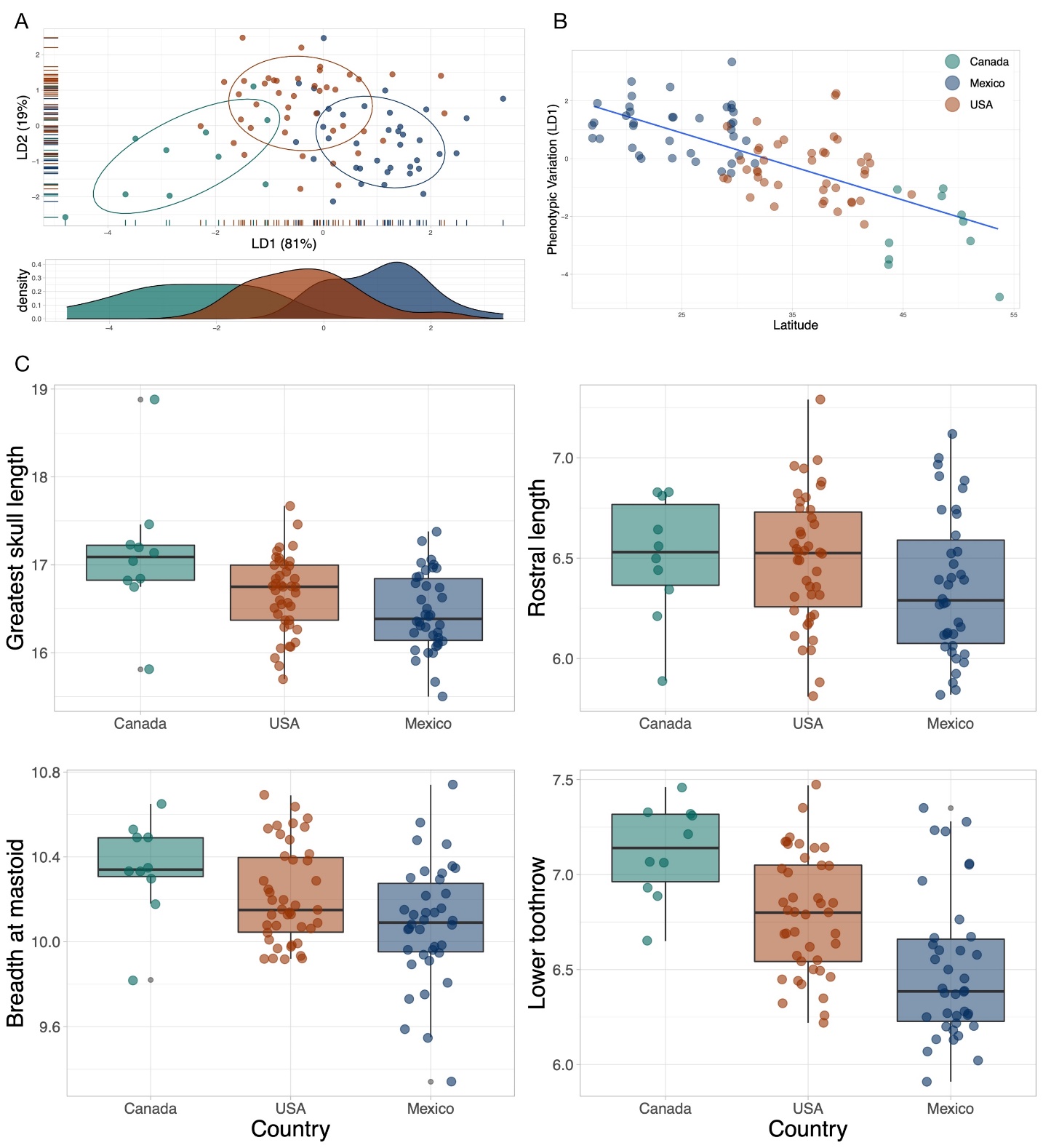


Supplementary Figure S4: Multiple Linear Regression result plots for hoary bats examining the relationship between phenotypic variability and three environmental features (A–C), elevation (D), latitude (E), and longitude (F). Multiple R^2^ = 0.585, adjusted R^2^ = 0.566 (F = 31.27 on 6 and 13 DF, *P* < 2.2e-16). Individual model results listed in Table 3 of main article. In each plot, black circles = *L. cinereus*, blue circles = *L. semotus*, and brown circles = *L. villosissimus*. Plot G shows the variance inflation factors (VIF) for each variable used; the dashed line represents a VIF threshold value of five; variables below this threshold show low correlation.


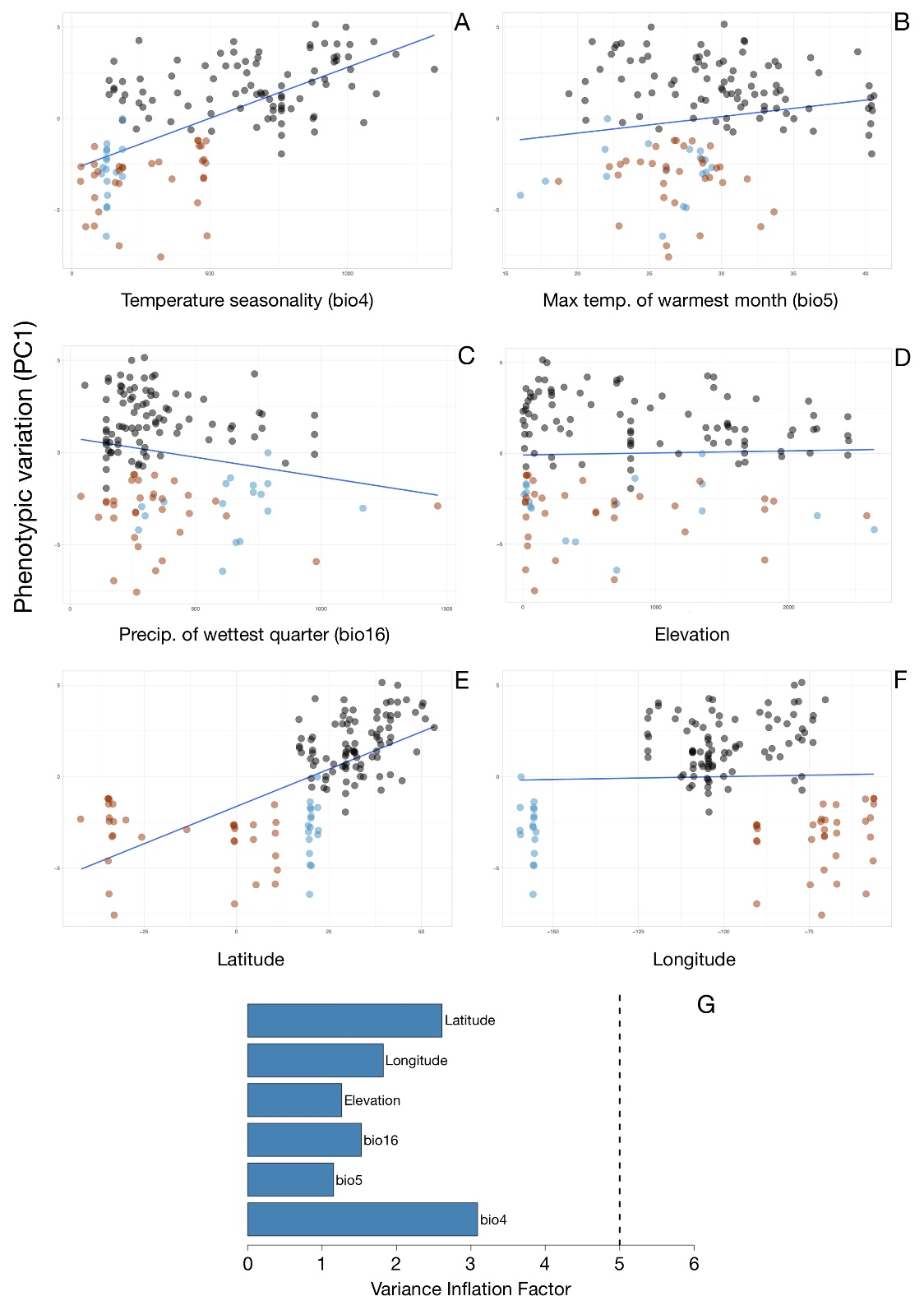


Supplementary Figure S5. Correlation matrix and heatmap of environmental variables used in phenotype-environment and niche deconstruction analyses. Fourteen WorldClim variables (www.worldclim.org) exceeding the Pearson Correlation coefficient of 0.75 were excluded from analyses. Variables retained included: bio4 = Temperature Seasonality, bio5 = Max Temperature of Warmest Month, bio15 = Precipitation Seasonality, bio16 = Precipitation of Wettest Quarter, bio17 = Precipitation of Driest Quarter, and bioalt = elevation.


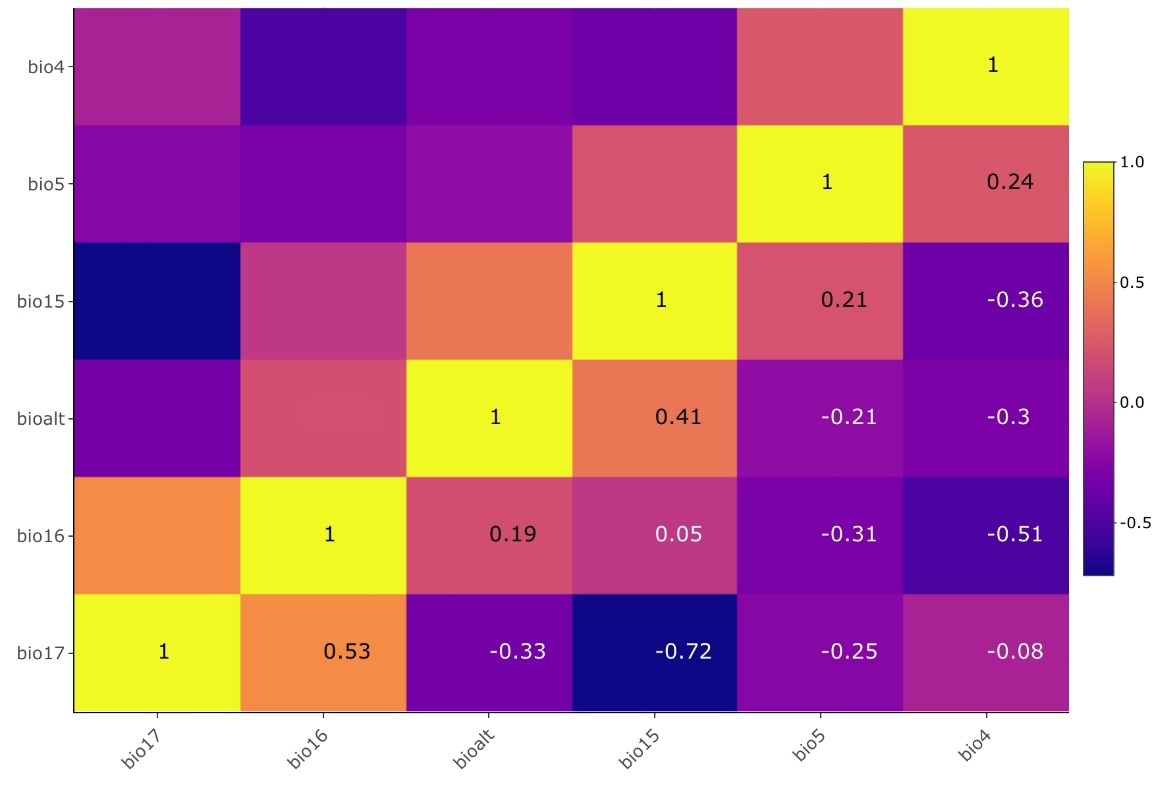


Table S1. Description of morphological characters measured in hoary bats. Additional details about each character can be obtained from the original sources. LD1 Coefficients refers to the uncorrelated coefficients for Linear Discriminant 1.

| **Character Description** | **Abbreviation** | **Source** | **LD1 Coefficients** |
| --- | --- | --- | --- |
| *Cranial* |  |  |  |
| Greatest Skull Length | GSL | Freeman (1981)^1^ | -0.0239 |
| Rostral Length | ROST LEN | Freeman (1981)^1^ | -0.0266 |
| Lacrimal Width | LACR W | Freeman (1981)^1^ | -0.6884 |
| Postorbital Width | POSTORB W | Freeman (1981)^1^ | 0.2569 |
| Breadth at Mastoids | MASTOID | Freeman (1981)^1^ | -0.9285 |
| Width at Upper Canines | C1 C1 | Freeman (1981)^1^ | -0.8281 |
| Width at Upper Molars | M3 M3 | Freeman (1981)^1^ | 0.1369 |
| Length of Glenoid Fossa | GLENO L | Freeman (1981)^1^ | 0.2204 |
| Length of maxillary tooth row | MAX TOOTH | Jacobs (1996)^2^ | -0.4180 |
| *Dentary* |  |  |  |
| Dentary Length | DEN L | Freeman (1981)^1^ | -0.2484 |
| Lower Toothrow | LOW TR | Freeman (1981)^1^ | 0.2497 |
| Moment Arm of Temporal | MOM1 COR | Freeman (1981)^1^ | 0.1758 |
| Moment Arm of Masseter | MOM2 ANG | Freeman (1981)^1^ | -0.2174 |
| Dentary Thickness | DEN TH | Freeman (1981)^1^ | 0.0353 |
| Length of Condyle | CON L | Freeman (1981)^1^ | 0.1088 |
| Length of masseter muscle scar | MASS MUSC | Jacobs (1996)^2^ | -0.2263 |

1. Freeman, P. W. (1981). A multivariate study of the family Molossidae (Mammalia, Chiroptera): morphology, ecology, evolution. *Fieldiana* 7, 1–168.
2. Jacobs, D. S. (1996). Morphological divergence in an insular bat, Lasiurus cinereus semotus. *Funct. Ecol.* 10, 622–630.

Table S2. Summary of cranial measurements for specimens of *Lasiurus cinereus*, *L. semotus*, and *L. villosissimus*. Numbers represent mean, minimum, and maximum measurements in mm. Description of morphological characters measured listed in Table S3.

| **Character Description** | ***L. cinereus*** | ***L. semotus*** | ***L. villosissimus*** |
| --- | --- | --- | --- |
| Greatest Skull Length | 2.812 (2.741–2.938) | 2.741 (2.705–2.771) | 2.728 (2.653–2.774) |
| Rostral Length | 1.863 (1.760–1.987) | 1.793 (1.708–1.893) | 1.746 (1.629–1.844) |
| Lacrimal Width | 2.158 (2.014–2.244) | 2.024 (1.953–2.079) | 2.048 (1.952–2.107) |
| Postorbital Width | 1.656 (1.556–1.732) | 1.606 (1.550–1.654) | 1.658 (1.564–1.716) |
| Breadth at Mastoids | 2.320 (2.234–2.374) | 2.230 (2.132–2.270) | 2.253 (2.197–2.289) |
| Width at Upper Canines | 1.482 (1.399–1.617) | 1.345 (1.238–1.416) | 1.354 (1.125–1.435) |
| Width at Upper Molars | 1.647 (1.530–1.892) | 1.538 (1.468–1.607) | 1.546 (1.281–1.619) |
| Length of Glenoid Fossa | 0.635 (0.512–0.797) | 0.575 (0.463–0.636) | 0.571 (0.418–0.727) |
| Length of maxillary tooth row | 1.609 (1.502–1.710) | 1.531 (1.433­–1.601) | 1.529 (1.435–1.595) |
| Dentary Length | 2.532 (0.431–2.634) | 2.503 (2.446–2.556) | 2.471 (2.376–2.531) |
| Lower Toothrow | 1.902 (1.777–2.011) | 1.859 (1.773–1.944) | 1.818 (1.728–1.923) |
| Moment Arm of Temporal | 1.337 (1.109–1.461) | 1.318 (1.256–1.376) | 1.238 (1.122–1.656) |
| Moment Arm of Masseter | 1.083 (0.936–1.217) | 0.984 (0.879–1.078) | 0.976 (0.824–1.081) |
| Dentary Thickness | 0.629 (0.285–0.832) | 0.506 (0.329–0.693) | 0.553 (0.300–0.693) |
| Length of Condyle | 0.578 (0.425–0.741) | 0.516 (0.307–0.625) | 0.473 (0.215–0.604) |
| Length of masseter muscle scar | 1.435 (1.235–1.562) | 1.374 (1.303–1.440) | 1.354 (1.244–1.466) |

Table S3. Genbank accession numbers for hoary bat specimens (*Lasiurus cinereus*, *L. semotus*, and *L. villosissimus*) used in genetic analysis.

| **Species** | **Museum No.** | **Genbank No.** | **Genes** |
| --- | --- | --- | --- |
| *L. cinereus* | AK11006 | MF990053.1, MF990138.1 | COI, RAG2 |
| *L. cinereus* | AK11013 | MF990054.1, MF990140.1 | COI, RAG2 |
| *L. cinereus* | AK11097 | MF990055.1, MF990142.1 | COI, RAG2 |
| *L. cinereus* | AK11210 | MF990056.1, MF990144.1 | COI, RAG2 |
| *L. cinereus* | AK11212 | MF990057.1, MF990146.1 | COI, RAG2 |
| *L. cinereus* | ASK1079 | MF990058.1, MF990148.1 | COI, RAG2 |
| *L. cinereus* | ASK3520 | MF990059.1, MF000149.1 | COI, RAG2 |
| *L. cinereus* | ASK4288 | KR350014.1, KR350135.1 | COI, RAG2 |
| *L. cinereus* | BPBM185003 | MF990062.1, MF990151.1 | COI, RAG2 |
| *L. cinereus* | BPBM185519 | MF990066.1, MF990155.1 | COI, RAG2 |
| *L. cinereus* | BPBM185539 | MF990068.1, MF990157.1 | COI, RAG2 |
| *L. cinereus* | CM82018 | KR350019.1, KR350137.1 | COI, RAG2 |
| *L. cinereus* | FJB27 | KR350046.1, KR350091.1 | COI, RAG2 |
| *L. cinereus* | FJB61 | KR350075.1, KR350127.1 | COI, RAG2 |
| *L. cinereus* | FJB62 | KR350076.1, KR350129.1 | COI, RAG2 |
| *L. cinereus* | LSUM368 | KR350081.1, KR350139.1 | COI, RAG2 |
| *L. cinereus* | MVZ199246 | KR350082.1, KR350141.1 | COI, RAG2 |
| *L. cinereus* | NK3562 | MF990071.1, MF990159.1 | COI, RAG2 |
| *L. cinereus* | NK3580 | MF990073.1, MF990162.1 | COI, RAG2 |
| *L. cinereus* | NK3599 | MF990074.1, MF990163.1 | COI, RAG2 |
| *L. cinereus* | NK3627 | MF990075.1, MF990164.1 | COI, RAG2 |
| *L. cinereus* | NK3642 | MF990076.1, MF990166.1 | COI, RAG2 |
| *L. cinereus* | NK36563 | MF990072.1, MF990160.1 | COI, RAG2 |
| *L. cinereus* | NK6534 | MF990077.1, MF990168.1 | COI, RAG2 |
| *L. cinereus* | NK8096 | MF990078.1, MF990170.1 | COI, RAG2 |
| *L. cinereus* | NK9191 | MF990079.1, MF990172.1 | COI, RAG2 |
| *L. cinereus* | NK9250 | MF990080.1, MF990174.1 | COI, RAG2 |
| *L. cinereus* | NK9273 | MF990081.1, MF990175.1 | COI, RAG2 |
| *L. semotus* | BPBM178452 | MF990060.1, MF990150.1 | COI, RAG2 |
| *L. semotus* | BPBM185245 | MF990063.1, MF990152.1 | COI, RAG2 |
| *L. semotus* | BPBM185478 | MF990064.1, MF990153.1 | COI, RAG2 |
| *L. semotus* | BPBM185479 | MF990065.1, MF990154.1 | COI, RAG2 |
| *L. semotus* | BPBM185538 | MF990067.1, MF990156.1 | COI, RAG2 |
| *L. semotus* | BPBM185540 | MF990069.1, MF990158.1 | COI, RAG2 |
| *L. semotus* | FJB35 | KR350054.1, KR350093.1 | COI, RAG2 |
| *L. semotus* | FJB37 | KR350056.1, KR350095.1 | COI, RAG2 |
| *L. semotus* | FJB41 | KR350060.1, KR350097.1 | COI, RAG2 |
| *L. semotus* | FJB42 | KR350061.1, KR350099.1 | COI, RAG2 |
| *L. semotus* | FJB44 | KR350062.1, KR350103.1 | COI, RAG2 |
| *L. semotus* | FJB45 | KR350063.1, KR350105.1 | COI, RAG2 |
| *L. semotus* | FJB46 | KR350064.1, KR350107.1 | COI, RAG2 |
| *L. semotus* | FJB47 | KR350065.1, KR350109.1 | COI, RAG2 |
| *L. semotus* | FJB49 | KR350066.1, KR350111.1 | COI, RAG2 |
| *L. semotus* | FJB52 | KR350067.1, KR350113.1 | COI, RAG2 |
| *L. semotus* | FJB53 | KR350068.1, KR350115.1 | COI, RAG2 |
| *L. semotus* | FJB55 | KR350069.1, KR350117.1 | COI, RAG2 |
| *L. semotus* | FJB57 | KR350071.1, KR350119.1 | COI, RAG2 |
| *L. semotus* | FJB58 | KR350072.1, KR350121.1 | COI, RAG2 |
| *L. semotus* | FJB59 | KR350073.1, KR350123.1 | COI, RAG2 |
| *L. semotus* | FJB60 | KR350074.1, KR350125.1 | COI, RAG2 |
| *L. semotus* | FJB63 | KR350077.1, KR350131.1 | COI, RAG2 |
| *L. semotus* | FJB64 | KR350078.1, KR350133.1 | COI, RAG2 |
| *L. villosissimus* | M260258 | KR350012.1 | COI |
| *L. villosissimus* | M268079 | KR350013.1 | COI |
| *L. villosissimus* | NK11502 | MF990082.1, MF990176.1 | COI, RAG2 |

Table S4. List of specimens studied for phenotypic analyses. AMNH = American Museum of Natural History; BM = Burke Museum of Natural History and Culture; EBD = Estación Biológica de Doñana, Sevilla; FMNH = Field Museum of Natural History; KU = University of Kansas Natural History Museum; LACM = Natural History Museum of Los Angeles County, MNCN = Museo Nacional de Ciencias Naturales, Madrid; MVZ = Museum of Vertebrate Zoology; ROM = Royal Ontario Museum; UF = Florida Museum of Natural History; USNM = United States National Museum. For details on each specimen, including georeferenced locality information, see: doi.org/10.5281/zenodo.6946172.

| **Genus** | **Species** | **Museum** | **Number** |
| --- | --- | --- | --- |
| *Lasiurus* | *cinereus* | AMNH | 1270 |
| *Lasiurus* | *cinereus* | AMNH | 17515 |
| *Lasiurus* | *cinereus* | AMNH | 27327 |
| *Lasiurus* | *cinereus* | AMNH | 137322 |
| *Lasiurus* | *cinereus* | AMNH | 166058 |
| *Lasiurus* | *cinereus* | AMNH | 176197 |
| *Lasiurus* | *cinereus* | AMNH | 205168 |
| *Lasiurus* | *cinereus* | AMNH | 207428 |
| *Lasiurus* | *cinereus* | AMNH | 208907 |
| *Lasiurus* | *cinereus* | AMNH | 258284 |
| *Lasiurus* | *cinereus* | AMNH | 258285 |
| *Lasiurus* | *cinereus* | EBD | 13022 |
| *Lasiurus* | *cinereus* | EBD | 13132 |
| *Lasiurus* | *cinereus* | FMNH | 10864 |
| *Lasiurus* | *cinereus* | FMNH | 140700 |
| *Lasiurus* | *cinereus* | FMNH | 172513 |
| *Lasiurus* | *cinereus* | FMNH | 172514 |
| *Lasiurus* | *cinereus* | FMNH | 195676 |
| *Lasiurus* | *cinereus* | KU | 40831 |
| *Lasiurus* | *cinereus* | KU | 44746 |
| *Lasiurus* | *cinereus* | KU | 73579 |
| *Lasiurus* | *cinereus* | KU | 73580 |
| *Lasiurus* | *cinereus* | KU | 73581 |
| *Lasiurus* | *cinereus* | KU | 73582 |
| *Lasiurus* | *cinereus* | KU | 73583 |
| *Lasiurus* | *cinereus* | KU | 73584 |
| *Lasiurus* | *cinereus* | KU | 73585 |
| *Lasiurus* | *cinereus* | KU | 73586 |
| *Lasiurus* | *cinereus* | KU | 73588 |
| *Lasiurus* | *cinereus* | KU | 80297 |
| *Lasiurus* | *cinereus* | KU | 82921 |
| *Lasiurus* | *cinereus* | KU | 92950 |
| *Lasiurus* | *cinereus* | KU | 98486 |
| *Lasiurus* | *cinereus* | KU | 98487 |
| *Lasiurus* | *cinereus* | KU | 98735 |
| *Lasiurus* | *cinereus* | KU | 98736 |
| *Lasiurus* | *cinereus* | KU | 98737 |
| *Lasiurus* | *cinereus* | KU | 100398 |
| *Lasiurus* | *cinereus* | LACM | 13835 |
| *Lasiurus* | *cinereus* | MNCN | 617 |
| *Lasiurus* | *cinereus* | MNCN | 618 |
| *Lasiurus* | *cinereus* | MNCN | 619 |
| *Lasiurus* | *cinereus* | MVZ | 55030 |
| *Lasiurus* | *cinereus* | MVZ | 80311 |
| *Lasiurus* | *cinereus* | MVZ | 80312 |
| *Lasiurus* | *cinereus* | MVZ | 93897 |
| *Lasiurus* | *cinereus* | MVZ | 114537 |
| *Lasiurus* | *cinereus* | MVZ | 121716 |
| *Lasiurus* | *cinereus* | MVZ | 124780 |
| *Lasiurus* | *cinereus* | MVZ | 139199 |
| *Lasiurus* | *cinereus* | MVZ | 139200 |
| *Lasiurus* | *cinereus* | MVZ | 199246 |
| *Lasiurus* | *cinereus* | MVZ | 225665 |
| *Lasiurus* | *cinereus* | ROM | 18458 |
| *Lasiurus* | *cinereus* | ROM | 22967 |
| *Lasiurus* | *cinereus* | ROM | 25996 |
| *Lasiurus* | *cinereus* | ROM | 29536 |
| *Lasiurus* | *cinereus* | ROM | 29537 |
| *Lasiurus* | *cinereus* | ROM | 32149 |
| *Lasiurus* | *cinereus* | ROM | 32291 |
| *Lasiurus* | *cinereus* | ROM | 36122 |
| *Lasiurus* | *cinereus* | ROM | 58731 |
| *Lasiurus* | *cinereus* | ROM | 58732 |
| *Lasiurus* | *cinereus* | ROM | 78358 |
| *Lasiurus* | *cinereus* | ROM | 78359 |
| *Lasiurus* | *cinereus* | ROM | 78360 |
| *Lasiurus* | *cinereus* | ROM | 78613 |
| *Lasiurus* | *cinereus* | ROM | 78614 |
| *Lasiurus* | *cinereus* | ROM | 78615 |
| *Lasiurus* | *cinereus* | ROM | 78616 |
| *Lasiurus* | *cinereus* | ROM | 78617 |
| *Lasiurus* | *cinereus* | ROM | 2441481 |
| *Lasiurus* | *cinereus* | ROM | 33620975 |
| *Lasiurus* | *cinereus* | UF | 11185 |
| *Lasiurus* | *cinereus* | UF | 13128 |
| *Lasiurus* | *cinereus* | UF | 31008 |
| *Lasiurus* | *cinereus* | USNM | 15345 |
| *Lasiurus* | *cinereus* | USNM | 38610 |
| *Lasiurus* | *cinereus* | USNM | 123816 |
| *Lasiurus* | *cinereus* | USNM | 178141 |
| *Lasiurus* | *cinereus* | USNM | 200373 |
| *Lasiurus* | *cinereus* | USNM | 218756 |
| *Lasiurus* | *cinereus* | USNM | 276572 |
| *Lasiurus* | *cinereus* | USNM | 329591 |
| *Lasiurus* | *cinereus* | USNM | 329597 |
| *Lasiurus* | *cinereus* | USNM | 347569 |
| *Lasiurus* | *cinereus* | USNM | 398462 |
| *Lasiurus* | *cinereus* | USNM | 448255 |
| *Lasiurus* | *cinereus* | USNM | 508889 |
| *Lasiurus* | *cinereus* | USNM | 508890 |
| *Lasiurus* | *semotus* | AMNH | 208651 |
| *Lasiurus* | *semotus* | AMNH | 276739 |
| *Lasiurus* | *semotus* | MVZ | 112947 |
| *Lasiurus* | *semotus* | MVZ | 113294 |
| *Lasiurus* | *semotus* | MVZ | 113386 |
| *Lasiurus* | *semotus* | MVZ | 113387 |
| *Lasiurus* | *semotus* | MVZ | 114344 |
| *Lasiurus* | *semotus* | MVZ | 114345 |
| *Lasiurus* | *semotus* | MVZ | 184549 |
| *Lasiurus* | *semotus* | ROM | 91239 |
| *Lasiurus* | *semotus* | USNM | 8545 |
| *Lasiurus* | *semotus* | USNM | 15630 |
| *Lasiurus* | *semotus* | USNM | 16138 |
| *Lasiurus* | *semotus* | USNM | 16139 |
| *Lasiurus* | *semotus* | USNM | 38983 |
| *Lasiurus* | *semotus* | USNM | 505291 |
| *Lasiurus* | *semotus* | USNM | 505292 |
| *Lasiurus* | *villosissimus* | AMNH | 15128 |
| *Lasiurus* | *villosissimus* | AMNH | 71356 |
| *Lasiurus* | *villosissimus* | AMNH | 268078 |
| *Lasiurus* | *villosissimus* | AMNH | 268079 |
| *Lasiurus* | *villosissimus* | AMNH | 268080 |
| *Lasiurus* | *villosissimus* | BM | 4171 |
| *Lasiurus* | *villosissimus* | BM | 5812 |
| *Lasiurus* | *villosissimus* | BM | 138161 |
| *Lasiurus* | *villosissimus* | BM | 138162 |
| *Lasiurus* | *villosissimus* | FMNH | 23629 |
| *Lasiurus* | *villosissimus* | FMNH | 42428 |
| *Lasiurus* | *villosissimus* | FMNH | 49153 |
| *Lasiurus* | *villosissimus* | FMNH | 51223 |
| *Lasiurus* | *villosissimus* | FMNH | 73664 |
| *Lasiurus* | *villosissimus* | FMNH | 73665 |
| *Lasiurus* | *villosissimus* | FMNH | 78685 |
| *Lasiurus* | *villosissimus* | LACM | 59566 |
| *Lasiurus* | *villosissimus* | MNCN | 620 |
| *Lasiurus* | *villosissimus* | MVZ | 118559 |
| *Lasiurus* | *villosissimus* | MVZ | 118560 |
| *Lasiurus* | *villosissimus* | MVZ | 145371 |
| *Lasiurus* | *villosissimus* | ROM | 35843 |
| *Lasiurus* | *villosissimus* | ROM | 50220 |
| *Lasiurus* | *villosissimus* | ROM | 76339 |
| *Lasiurus* | *villosissimus* | ROM | 76340 |
| *Lasiurus* | *villosissimus* | USNM | 15745 |
| *Lasiurus* | *villosissimus* | USNM | 200367 |
| *Lasiurus* | *villosissimus* | USNM | 240941 |
| *Lasiurus* | *villosissimus* | USNM | 307973 |
| *Lasiurus* | *villosissimus* | USNM | 370971 |
| *Lasiurus* | *villosissimus* | USNM | 370972 |
| *Lasiurus* | *villosissimus* | USNM | 387740 |
| *Lasiurus* | *villosissimus* | USNM | 441840 |
